# Reflex Nervous Action of the Uterus

**Published:** 1849-11

**Authors:** W. Tyler Smith

**Affiliations:** London


*Reflex Nervous Action of the Uterus.* By W. Tyler Smith, M. D., London. The Reflex actions of the uterus are very numerous, and it is upon these, and the numerous extra-uterine reflex actions excited during the process, that the natural performance of parturition essentially depends. Contraction of the uterus, from irritation of the mammæ, as in the act of suckling the child; contraction of this organ from the cold water douche, applied to the vulva or the abdominal surface; contraction excited by irritating the rectum, as by stimulating enemata; or of the stomach, by drinking a gulp of cold water; of the ovaria, by the presence of the menstrual nisus; of the vagina, by manual irritation, as in "taking a pain;" of the os uteri by irritation, as in the introduction of the hand into the uterus—are all to be considered as so many instances of reflex spinal action. Thus, in parturition, the uterus may be excited, in a reflex form, by irritation of the mammary incident excitor nerves; the pubic and abdominal branches of the intercostals; the rectal; the gastric division of the pneumogastric; the ovarian nerves; and also by the nerves of the vagina, and the os and cervix uteri.

Many of the different forms of abortion—particularly when the causes are extra uterine—strikingly illustrate the reflex action of the uterus. A series of cases of abortion would be one of the best expositions of reflex uterine action. Abortion may be caused by irritation of the mammæ, from the sucking of an infant, after milk had ceased to be secreted, as in cases in which the mother becomes pregnant during lactation; abortion may be excited, as a morbid reflex act, from irritation of the bladder, by a calculus; by irritation of the tri-facial nerve, as in cutting the dens sapienta; by the mechanical irritation of coitus; by plugging the vagina; by disease of the os and cervix uteri—malignant or simple induration, inflammation and ulceration; by the irritation of the placenta attached within the uterine mouth; by ovarian irritation in ovarian disease; by irritation of the rectum, as from ascarides, and the use of irritating purgatives or enemata; by puncturing the membranes, and evacuating the liquor amnii, so as to bring the head of the fetus to act as an excitant to the os uteri; by irritation of the inner surface of the uterus itself, in cases of blighted fetus, where the ovum acts as a foreign body; by riding on horseback, or any other violent exercise calculated, by succussion, to bring the head of the fetus into violent contact with the os uteri; and by other sources of irritation to incident spinal nerves which might be enumerated. All these are so many instances of uterine reflex action, the distant parts of the economy being brought into connection with the uterus through the medium of the spinal marrow and its special incident excitor and reflex motor nerves.—These facts are of most extensive practical application in devising means for the prevention of abortion.

In cases of abortion in which the irritation is applied to the os or cervix uteri, or the internal surface of the organ, the immediate action depending on the irritability of the uterus itself is called forth, in connection with true reflex action; but in the instances in which a distal organ is irritated, there can be no doubt whatever of the purely reflex nature of the uterine action which ensues."

